# Antibiotic Consumption Patterns in European Countries Might Be Associated with the Prevalence of Type 1 and 2 Diabetes

**DOI:** 10.3389/fendo.2022.870465

**Published:** 2022-05-06

**Authors:** Gábor Ternák, Márton Németh, Martin Rozanovic, Lajos Bogár

**Affiliations:** ^1^ Medical School, Institute of Migration Health, University of Pécs, Pécs, Hungary; ^2^ Department of Anesthesiology and Intensive Care, Medical School, University of Pécs, Pécs, Hungary

**Keywords:** diabetes type 1 (T1D), diabetes type 2 (T2D), antibiotics, antibiotic classes, microbiome, dysbiosis, prevalence, concordance

## Abstract

Several publications have raised the issue that the development of diabetes precedes the alteration of the microbiome (dysbiosis) and the role of environmental factors. Antibiotic use induces dysbiosis, and we wanted to estimate the associations between the consumption of antibiotics and the prevalence of diabetes (both types 1 and 2; T1D and T2D, respectively) in European countries. If such an association exists, the dominant use antibiotic classes might be reflected in the prevalence rates of T1D and T2D in different countries. Comparisons were performed between the prevalence of diabetes estimated for 2019 and featured in the Diabetes Atlas and the average yearly consumption of antibiotic classes between 2010 and 2109, calculated from the European Centre for Disease Prevention and Control (ECDC) yearly reports on antibiotic consumption in Europe. Pearson’s correlation and variance analyses were used to estimate the possible relationship. Strong positive (enhancer) associations were found between the prevalence of T1D and the consumption of tetracycline (J01A: *p* = 0.001) and the narrow-spectrum penicillin (J01CE: *p* = 0.006; CF: *p* = 0.018). A strong negative (inhibitor) association was observed with broad-spectrum, beta-lactamase-resistant penicillin (J01CR: *p* = 0.003), macrolide (J01F: *p* = 0.008), and quinolone (J01M: *p* = 0.001). T2D showed significant positive associations with cephalosporin (J01D: *p* = 0.048) and quinolone (J01M: *p* = 0.025), and a non-significant negative association was detected with broad-spectrum, beta-lactamase-sensitive penicillin (J01CA: *p* = 0.067). Countries showing the highest prevalence rates of diabetes (top 10) showed concordance with the higher consumption of “enhancer” and the lower consumption of “inhibitor” antibiotics (top 10), as indicated by variance analysis. Countries with high prevalence rates of T1D showed high consumption of tetracycline (*p* = 0.015) and narrow-spectrum, beta-lactamase sensitive penicillin (*p* = 0.008) and low consumption of “inhibitor” antibiotics [broad-spectrum, beta-lactamase-resistant, combination penicillin (*p* = 0.005); cephalosporin (*p* = 0.036); and quinolone (*p* = 0.003)]. Countries with high prevalence rates of T2D consumed more cephalosporin (*p* = 0.084) and quinolone (*p* = 0.054) and less broad-spectrum, beta-lactamase-sensitive penicillin (*p* = 0.012) than did other countries. The development of diabetes-related dysbiosis might be related to the higher consumption of specific classes of antibiotics, showing positive (enhancer) associations with the prevalence of diabetes, and the low consumption of other classes of antibiotics, those showing negative (inhibitory) associations. These groups of antibiotics are different in T1D and T2D.

## Introduction

Type 1 and 2 diabetes (T1D and T2D, respectively) are chronic diseases that develop either when the pancreas could not produce a sufficient amount of insulin or when the body cannot utilize the insulin it produces. Hyperglycemia is a common result of uncontrolled diabetes that leads to serious damage to several organs, particularly the nervous system, blood vessels, and kidneys. Diabetes is one of the biggest public health problems worldwide, imposing a heavy global burden on health services (1).

According to recent data, approximately 60 million people in Europe are suffering from diabetes, which is 6.3% of the population (age-adjusted) in Europe. Worldwide, 1 in 11 adults (20–79 years old, 463 million) suffers from diabetes. Europe has the largest number of youngsters with T1D, 296,500 in total (2).

Apart from specific categories (gestational, diseases of the exocrine pancreas, and drug- or chemical-induced diabetes), two major types of diabetes mellitus could be classified as T1D and T2D. T1D is characterized by the autoimmune destruction of insulin-producing β-cells, which usually leads to absolute insulin deficiency. On the other hand, T2D is due to a progressive loss of adequate β-cell insulin production based on the background of insulin resistance (3).

The incidence and prevalence of T1D have considerably increased in the past 30 years, probably due to changes in the environment that could not be appropriately identified yet. The pathomechanisms leading to the development of T1D cannot be fully explained only by genetic background, but external factors might play a part in the development of T1D as well (4, 5).

T1D is considered an autoimmune disease, developing as the result of T-cell-mediated beta cell destruction of the pancreas in genetically susceptible individuals. Several susceptible genes and loci have been found to play a part in the development of T1D. Human leukocyte antigen (HLA) regions are considered to contribute about 50% of the genetic susceptibility. External factors may change the expression of the genes *via* epigenetic mechanisms and promote the development of T1D in genetically susceptible individuals, but this pathomechanism is not appropriately understood. The development of T1D starts early in life, and the destruction of insulin-producing beta cells and the lack of endogenous insulin cause a life-long need for exogenous insulin therapy (6–8).

In recent years, the emergence of high-throughput sequencing has allowed analyzing the role of the gut microbiota in the development of T1D. Significant changes in the composition of the gut microbiome (dysbiosis) have been found in subjects with clinical or preclinical T1D. However, whether the dysbiosis is a cause or a consequence of the disease remains unclear, but increasing evidence has supported a causal link between the altered intestinal microflora and T1D development. Perturbation of the ecological balance of the gut microbiome, commonly due to antibiotic use, can cause and exacerbate diseases (9–12).

T2D is characterized by the imbalance of the blood glucose level, and it accounts for 90% of all cases of diabetes. It is considered a frequently detected metabolic disorder that is associated with an altered lipid profile, obesity, and high blood pressure. Genetic factors, a high-energy diet, and the lack of physical activity are considered major risk factors in the development of T2D. Several studies have indicated the presence of an altered gut flora as a factor in the rapid progression of insulin resistance. In T2D, during the initial phase of the disease, hyperglycemia develops as the result of the inability of the body’s tissues (cells) to respond fully to insulin, what we call “insulin resistance.” During this period of insulin resistance, the hormone is ineffective, and it induces more insulin production until the beta cells can keep up with the demand before exhaustion. T2D most commonly occurs in older adults, but is increasingly seen in children and younger adults owing to the rising levels of obesity, physical inactivity, and inappropriate diet (13). In one study, quantitative PCR analysis indicated that the gut microbial composition in patients with T2D was partially different from that in healthy individuals. Certain taxa of microbiomes, such as *Faecalibacterium prausnitzii*, were found to be significantly lower in patients with T2D (*p* = 0.038), and *Bacteroides fragilis* was underrepresented in the microbiota of the group with diabetes, but this difference between the two groups was not significant (14–16).

## Concept

In one of our previous studies (17), we have observed the inverse association between the prevalence of T1D and the utilization of broad-spectrum, beta-lactamase-resistant, combination penicillin (J01CR) in European countries, while the outstanding utilization of narrow-spectrum penicillin (J01CE, CF), particularly in Scandinavian countries, showed a correlation with the higher prevalence of T1D.

This present work intended to confirm the above association by using a slightly modified antibiotic consumption database. We also wanted to elucidate the probable role of the different antibiotic classes in the development of T1D and T2D.

Based on the above observations, we hypothesized that different classes of antibiotics inducing different types of dysbiosis might influence the development of diabetes (T1D and T2D). The altered microbiome might generate diverse mediator molecules, which could act as enhancers or inhibitors, through different mechanisms, in addition to other factors, in the process of developing diabetes. It was suspected that if such an association exists, the antibiotic consumption patterns might influence the prevalence of T1D and T2D in different countries.

## Materials and Methods

The average yearly antibiotic consumption data have been compared to the diabetes prevalence figures in order to estimate the positive and negative correlations between consumption of different classes of antibiotics and the prevalence of T1D and T2D.The average yearly antibiotic consumption in 2010–2019 was calculated for 30 European countries published in the European Centre for Disease Prevention and Control (ECDC) database (18) and expressed in defined daily dose per 1,000 inhabitants per day (DID) at Anatomical Therapeutic Chemical Classification System (ATC) level 3 for the five major antibiotic classes: tetracycline (J01A), penicillin (J01C), cephalosporin (J01D), macrolides (J01F), and quinolones (J01M). Subgroups of penicillin (J01C) were separately calculated at ATC level 4, as indicated in the ECDC database: narrow-spectrum beta-lactamase-sensitive penicillin (J01CE), beta-lactamase-resistant narrow-spectrum penicillin (J01CF), broad-spectrum beta-lactamase sensitive penicillin (J01CA), and broad-spectrum penicillin combined with a beta-lactamase inhibitor (J01CR). The antibiotic classes included in the study have been selected as the most frequently consumed antibiotics in the community, covering over 90% of the total antibiotic consumption for systemic use (92.4%). Rarely indicated antibiotics (sulfonamides, aminoglycosides, etc.) were not included.

Data of the average yearly antibiotic consumption of the five major antibiotic classes (ATC level 3) and the penicillin subgroups have been estimated as a relative average share in percent of the total amount of the systemic antibiotic consumption (J01, in DID as 100%) in the European countries (*n* = 30) included in the study.

Diabetes prevalence data for comparison have been extracted from the Diabetes Atlas for 2019 (19). The prevalence of T1D in 0- to 19-year-olds was calculated for 100,000 inhabitants per country. As for adult diabetes, the figures for age-adjusted (20–79 years) prevalence have been similarly calculated and used for comparison.

The rank order of countries (reducing) starting with the highest prevalence rates of T1D and T2D (top 10) was compared to the rank order of the utilization of different antibiotic classes to estimate the possible concordance or discordance between countries with higher consumption of “enhancer” antibiotic classes or low consumption of “inhibitor” types of antibiotics.

### Statistics

Pearson’s correlations were used to calculate the possible significance between the antibiotic consumption data of the major antibiotic classes and the prevalence of T1D and T2D. Positive significance was estimated if the correlation (*r*) was a positive number and the *p*-value was ≤0.05, considered as a positive “enhancer” association. Negative significance was estimated when the correlation (*r*) was a negative number and the related *p*-value was ≤0.05. This result was counted as a negative, non-supportive “inhibitor” association. A non-significant correlation was considered when the *p*-value falls between 0.05 and 0.09.

Variance analyses (ANOVA) were applied to estimate the association (concordance) between the consumption of “enhancer” and “inhibitor” types of antibiotics and countries with the top highest prevalence rates of T1D and T2D.

Logistic regression analysis was performed to estimate the odds ratio (OR) at 95% confidence interval (CI) together with the related *p*-values, which are shown in **Table 1**.

**Table 1:** Average yearly antibiotic consumption 2010–2019 estimated as a relative share in percentage of the total consumption of systemic antibiotics (J01), expressed as defined daily dose per 1,000 inhabitants per day (DID), compared to the prevalence of type 1 (T1D) and type 2 (T2D) diabetes for 100,000 population (2019).

Average antibiotic consumption for 2010–2019	Total systemic antibiotic consumption in DID (J01 100%) and relative share in %										People with diabetes (20–79 years), 100,000 population/country	Type 1 diabetes (0–19 years), 100,000 population/country
	J01	J01A	J01C	J01CA	J01CE	J01CF	J01CR	J01D	J01F	J01M		
Austria	12	7.41	39.91	6.58	6.58	0.08	26.75	12.91	24.83	10.16	**7,214.85**	**33.74**
Belgium	22.25	9.07	46.42	22.2	0.13	1.16	22.92	6.29	15.28	10.02	**4,887.57**	**35.71**
Bulgaria	17.87	9.62	30.38	17.51	1.06	0	11.08	19.13	20.42	14.6	**6,275.16**	**15.6**
Croatia	17.32	6.04	44.11	11.37	3.81	0	28.86	16.39	16.62	8.31	**5,078.91**	**31.28**
Cyprus	26.64	12.68	34.3	9.12	0.3	0.07	24.84	20.53	10.96	17.83	**7,719.05**	**33.63**
Czechia	16.64	12.68	35.81	6.97	11.17	0.3	17.3	11.17	22.05	5.7	**7,675.09**	**38.44**
Denmark	15.1	10.79	63.64	21.12	28.34	9.2	4.9	0.19	12.64	3.17	**6,467**.**17**	**53.89**
Estonia	10.25	15.41	32.29	16.78	1.85	0	13.65	10.73	23.02	8.09	**4,437.15**	**37.8**
Finland	15.73	24.72	30.38	16.84	7.88	0.08	5.4	13.54	7.18	4.83	**6,767.06**	**130.21**
France	23.54	13.55	52.54	31.18	0.72	1.06	19.75	8.62	13.89	6.92	**5,354.63**	**42.01**
Germany	12.97	16.26	26.44	17.27	6.26	0.07	1.87	21.74	17.88	9.63	**11,441.28**	**39.82**
Greece	31.19	7.98	30.65	14.17	0.25	0	16.25	24.39	24.04	8.43	**5,834.31**	**29.46**
Hungary	13.62	8.66	33.99	6.46	1.9	0	25.62	13.95	21.51	16.29	**7,051.25**	**36.05**
Iceland	19.03	25.27	47.45	16.97	10.93	5.45	14.13	3.04	8.46	4.78	**5,405.2**	**29.7**
Ireland	19.83	14.46	48.21	14.92	5.34	7.06	20.92	5.95	20.77	4.18	**3,075.52**	**68.48**
Italy	21.7	2.48	45.94	12.02	0	0.04	33.87	10.69	20.69	14.42	**6,052.39**	**26.39**
Latvia	11.14	20.46	38.5	25.94	0.44	0	12.11	4.8	15.43	8.88	**5,408.46**	**15.56**
Lithuania	13.9	10.57	46.97	35.25	1.65	0	10.14	8.63	14.31	6.83	**4,080.3**	**35.7**
Luxembourg	21.89	8.26	39.28	14.29	0.09	0.82	24.16	15.3	17.81	11.42	**4,733.18**	**33.1**
Malta	19.26	6.9	33.28	2.54	0.51	0.31	29.95	22.01	20.35	11.94	**6,702.58**	**33.1**
Netherlands	9.44	23.72	32.52	13.87	2.75	4.66	11.22	0.42	15.04	8.15	**5,973.78**	**42.79**
Norway	15.16	19.59	39.77	13.98	21.56	4.1	0.12	0.59	9.36	2.96	**5,477.75**	**71.19**
Poland	20.9	11	31.86	16.36	1.14	0.04	14.25	13.49	20	6.31	**6,182.76**	**33.23**
Portugal	17.65	4.87	47.81	9.63	0.11	3	35.07	9	16.94	12.12	**10,628.7**	**24.38**
Romania	25.76	4.19	47.47	17.97	3.1	2.52	23.83	30.59	11.29	12.92	**6,553.33**	**14.35**
Slovakia	19.92	8.08	29.41	5.02	6.07	0	18.32	22.99	26.6	9.73	**6,922.78**	**25.67**
Slovenia	11.72	3.49	59.47	19.36	14.33	1.42	24.4	2.81	15.35	9.38	**5,895.55**	**28.88**
Spain	19.78	5.2	55.3	21.94	0.45	1.06	31.95	9.4	12.28	12.79	**7,750.86**	**33.2**
Sweden	12.25	22.36	50.44	8.73	27.59	12.2	1.79	1.14	4.81	5.55	**5,226.82**	**86.24**
UK	17.18	27.35	38.12	20.43	4.77	8.38	4.54	1.86	17.05	2.61	**3,992.3**	**58.24**
Pearson’s correlation												
T1D, *R*	−0.226	**0.58**	−0.008	−0.014	**0.488**	**0.43**	*−0.519*	*−0.349*	*−0.473*	*−0.558*		
T1D, *P*	0.251	**0.001**	0.968	0.94	**0.006**	**0.018**	***0.003***	***0.059***	***0.008***	***0.001***		
T2D, *R*	−0.027	−0.242	−0.212	*−0.339*	−0.051	−0.277	0.167	**0.364**	0.065	0.41		
T2D, *P*	0.886	0.198	0.261	*0.067*	0.79	0.138	0.377	**0.048**	0.703	0.025		
T1D, OR	0.907	**1.270**	1.052	1.054	**1.174**	**1.951**	***0.840***	***0.746***	***0.800***	***0.416***		
T1D, 95% CI	0.758–1.084	**1.073–1.502**	0.965–1.148	0.943–1.177	**1.019–1.353**	**1.193–3.191**	***0.735–0.960***	***0.596–0.935***	***0.655–0.976***	***0.214–0.807***		
T1D, *P*	0.282	**0.005**	0.250	0.356	**0.027**	**0.008**	***0.011***	***0.011***	***0.028***	***0.010***		
T2D,OR	1.013	0.913	0.959	***0.868***	0.999	0.851	1.040	**1.165**	1.048	**1.297**		
T2D, 95% CI	0.880–1.168	0,811–1.028	0.885–1.040	***0.756–0.996***	0.910–1.097	0.648–1.117	0.964–1.122	**1.029–1.319**	0.912–1.203	**1.025–1.642**		
T2D, *P*	0.853	0.132	0.316	***0.044***	0.985	0.245	0.315	**0.016**	0.509	**0.030**		

Pearson’s correlation results are shown at the bottom of the table. Positive significance is displayed in bold, while negative significance is emphasized italics and underlined.

J01, antibiotics for systemic use; J01A, tetracycline; J01C, penicillin; J01CA, broad-spectrum, beta-lactamase-sensitive penicillin; J01CE, narrow-spectrum, beta-lactamase-sensitive penicillin; J01CE, narrow-spectrum, beta-lactamase-resistant penicillin; J01CR, broad-spectrum, beta-lactamase-resistant, combination penicillin; J01D, cephalosporin; J01F, macrolides; J01M, quinolone

**Figure 1:**
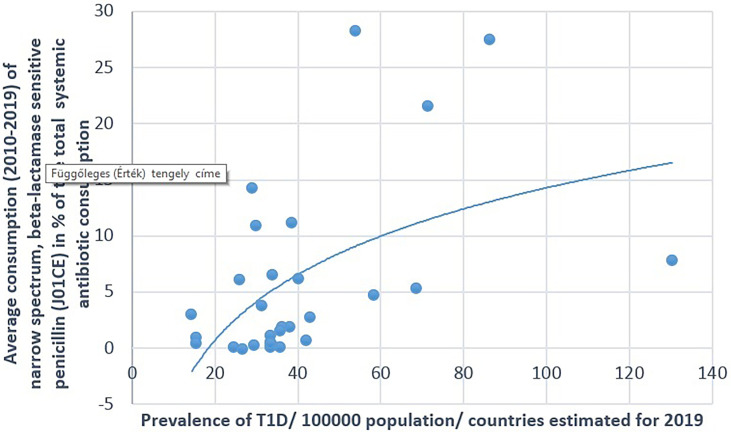
Significant positive association between the average (2010–2019) consumption of narrow-spectrum, beta-lactamase-sensitive penicillin and the prevalence of type 1 diabetes (T1D) (2019).

**Figure 2:**
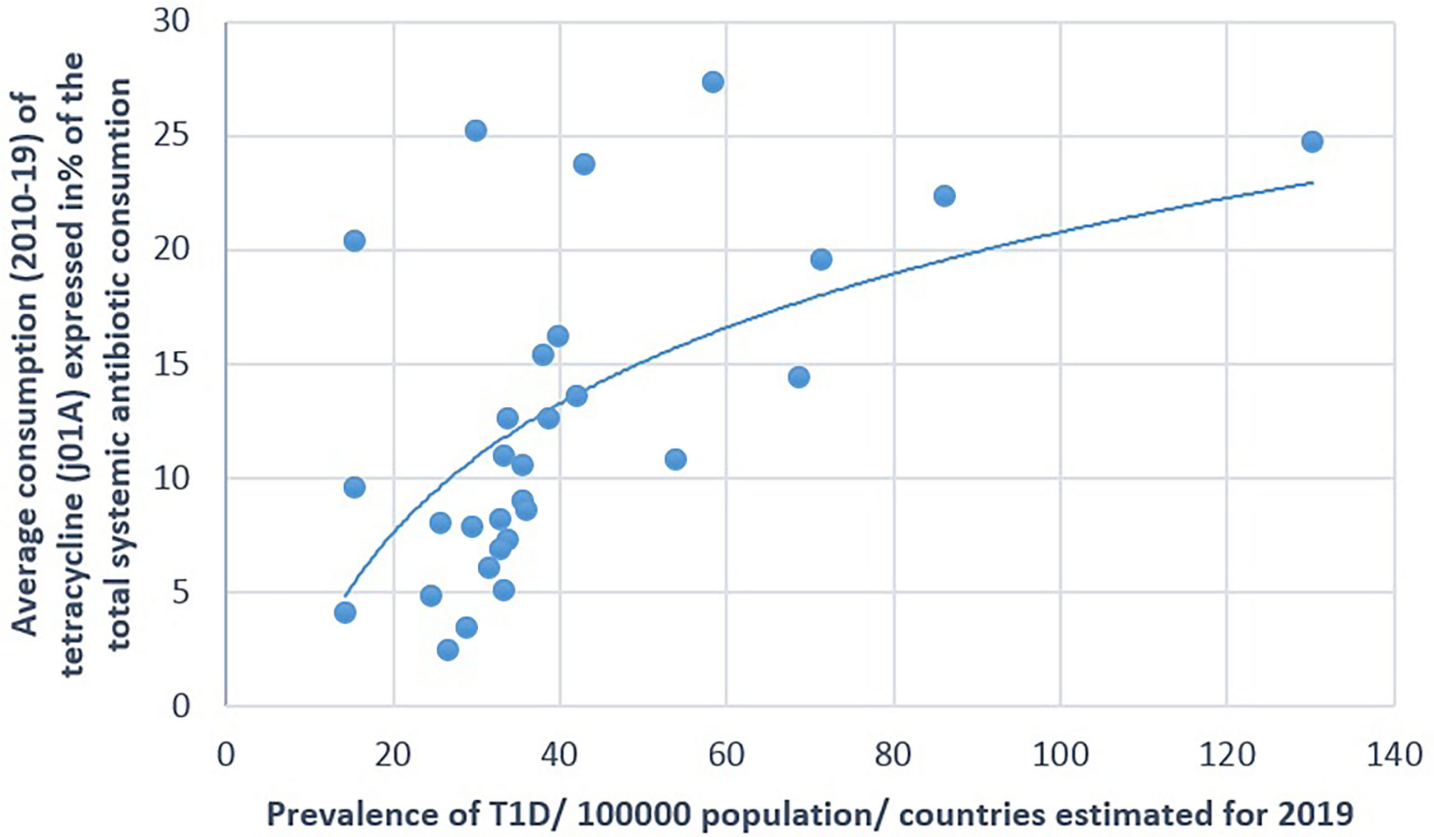
Significant positive association between the average consumption (2010–2019) of tetracycline (J01A) and the prevalence of type 1 diabetes (T1D) (2019).

**Figure 3:**
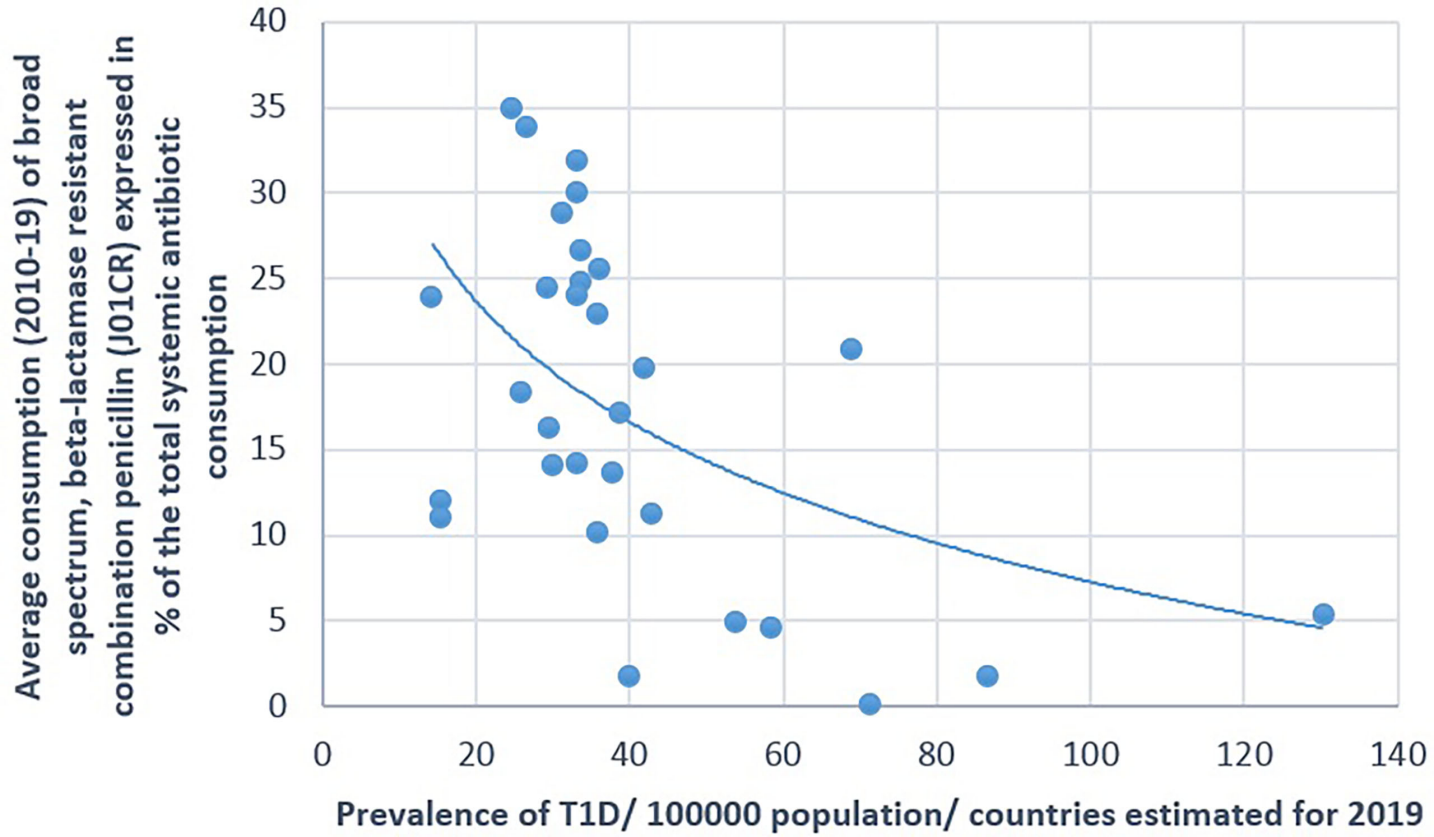
Significant negative association between the average consumption (2010–2019) of broad-spectrum, beta-lactamase-resistant, combination penicillin (J01CR) and the prevalence of type 1 diabetes (T1D) (2019).

**Figure 4:**
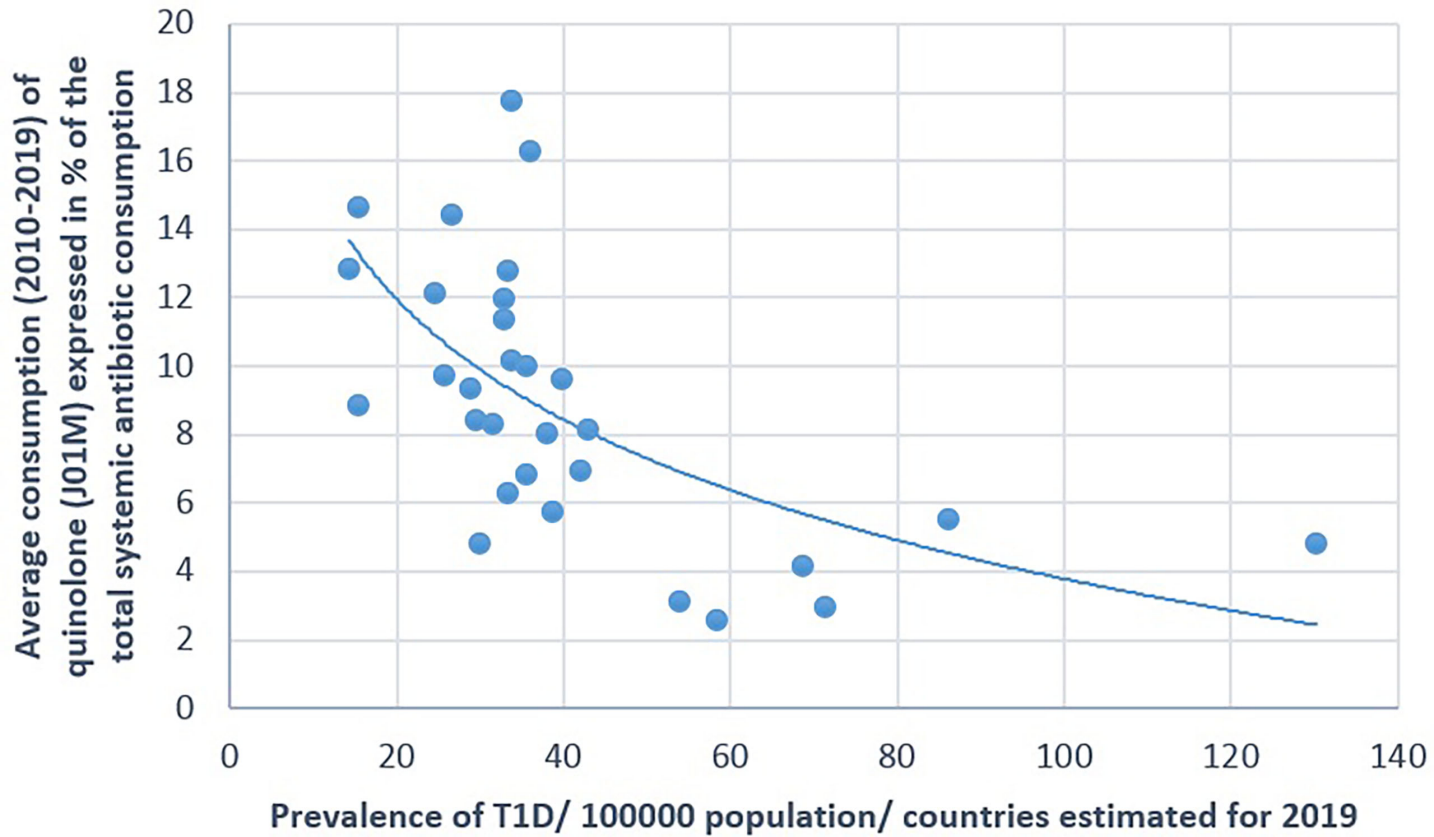
Significant negative association between the average consumption (2010–2019) of quinolone and the prevalence of type 1 diabetes (T1D) (2019).

**Figure 5:**
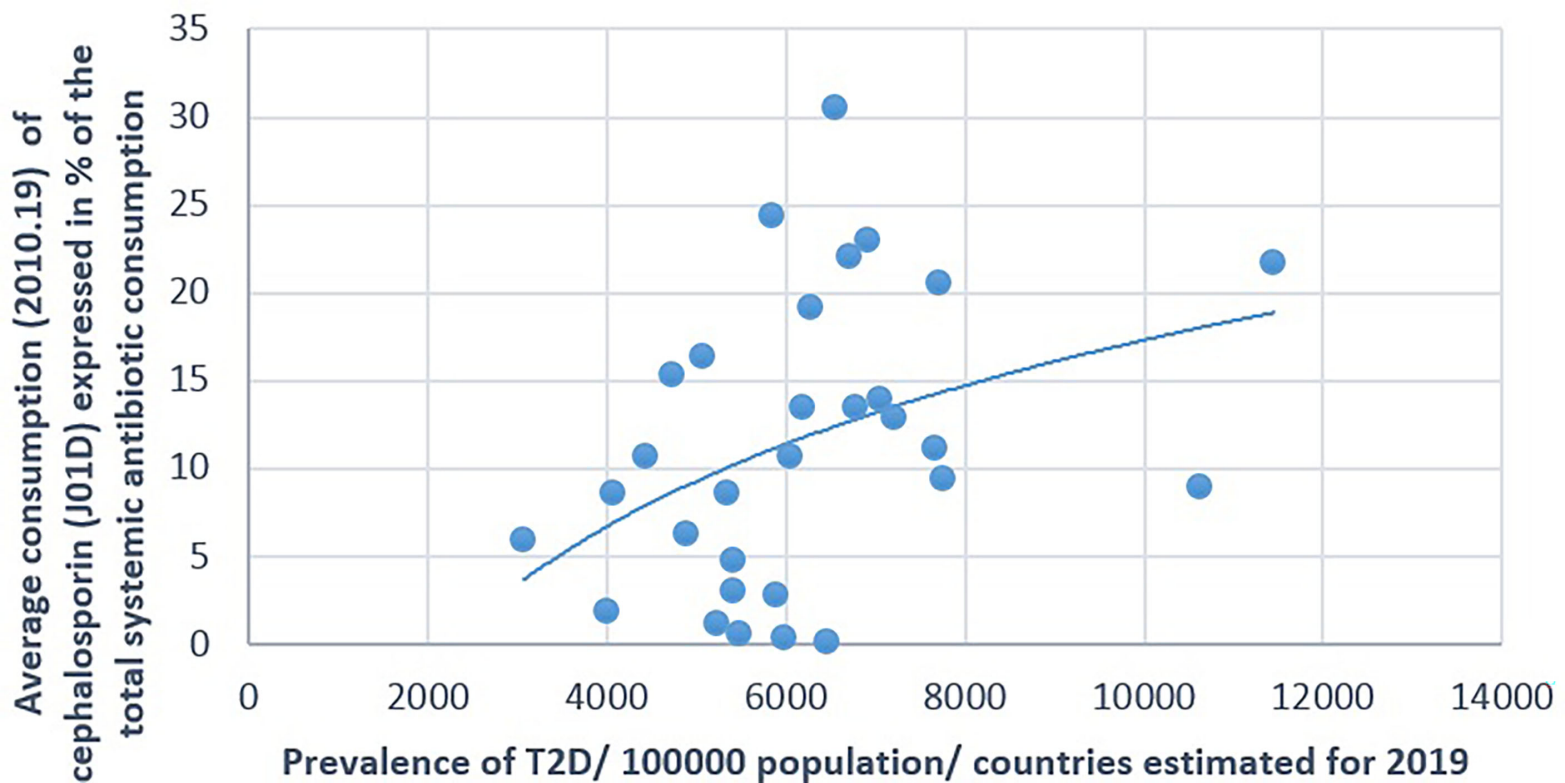
Significant positive association between the average consumption (2010–2019) of cephalosporin and the prevalence of type 2 diabetes (T2D) (2019).

**Figure 6:**
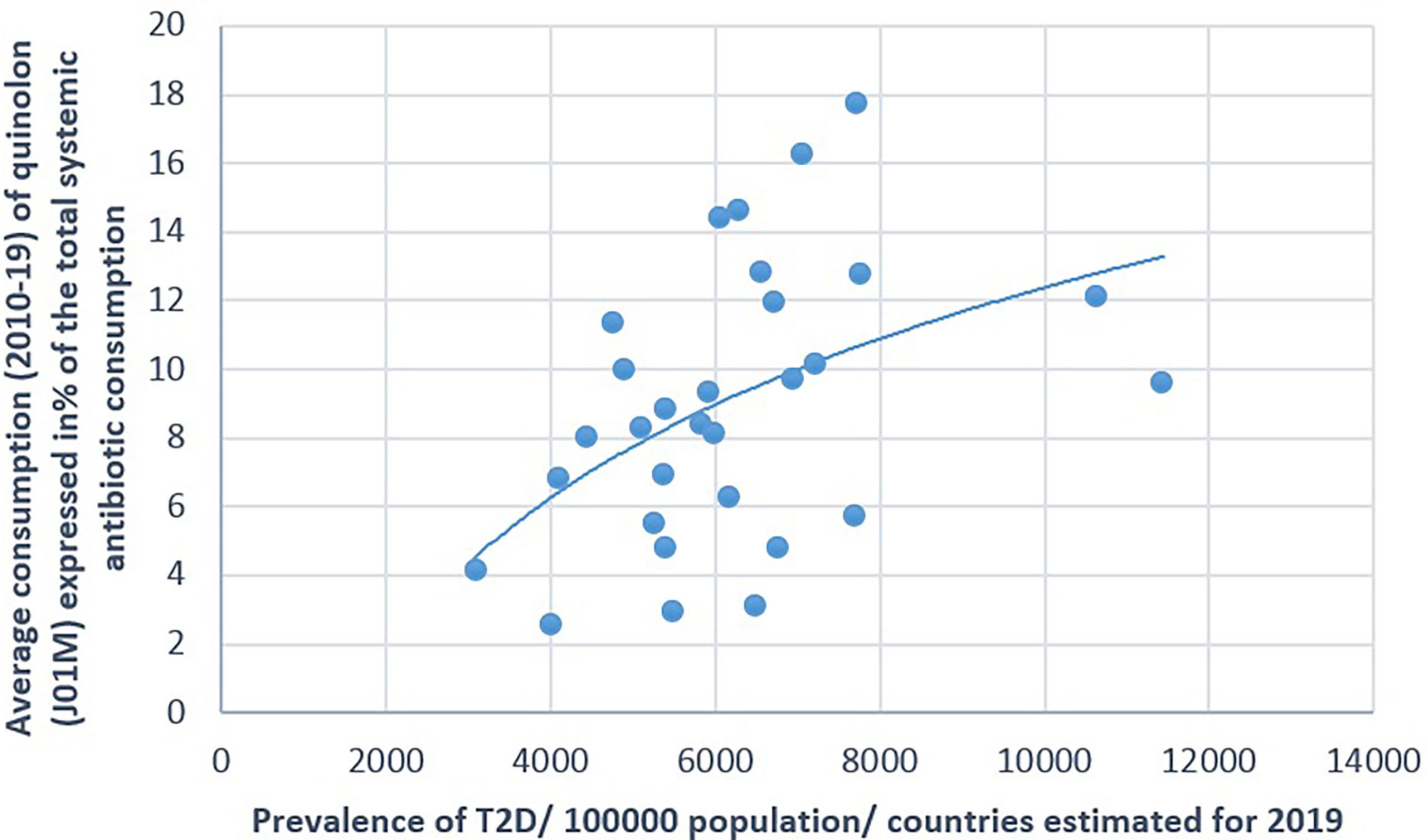
Significant positive association between the average consumption (2010–2019) of quinolone (J01M) and the prevalence of type 2 diabetes (T2D) (2019).

**Figure 7:**
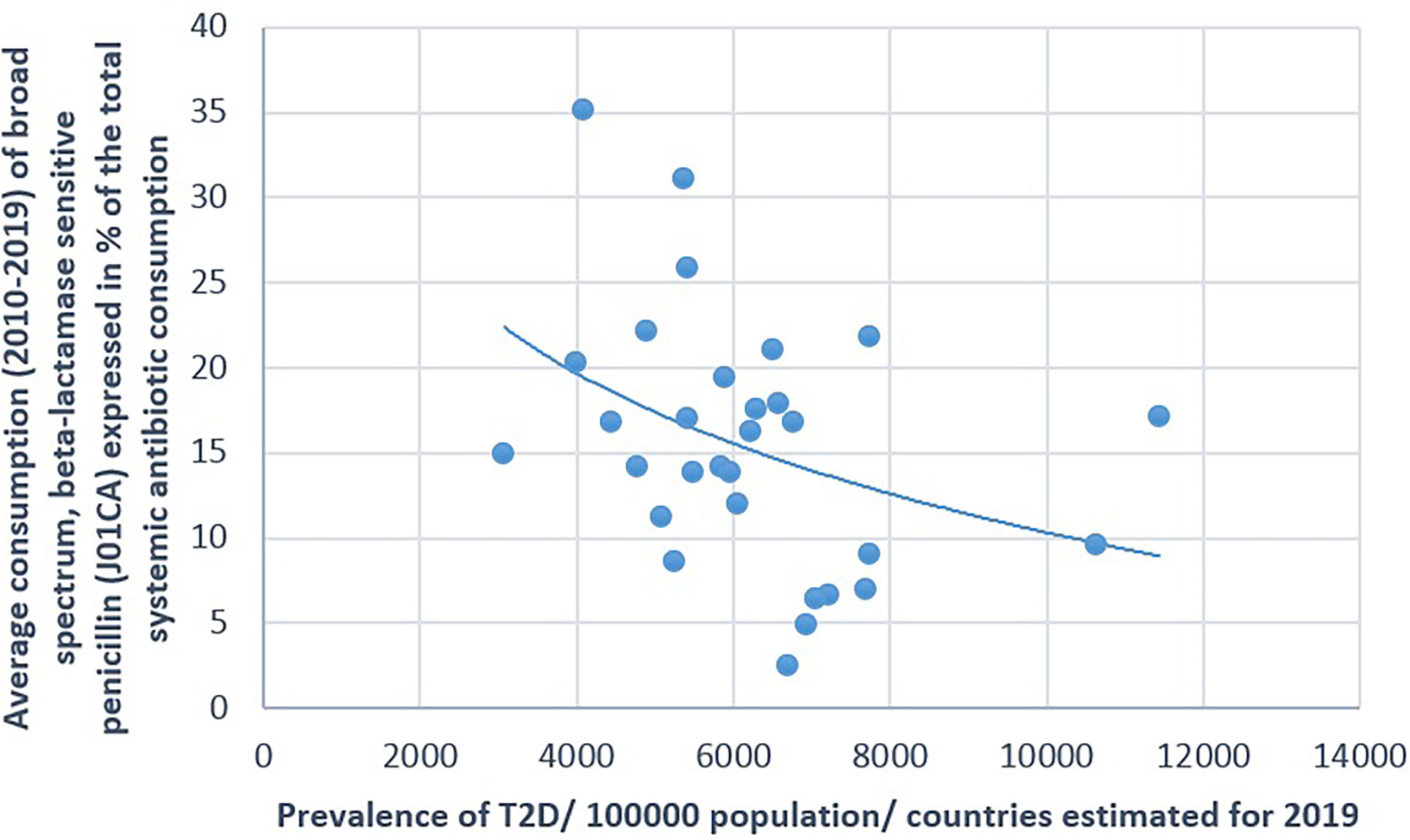
Significant negative association between the average consumption (2010–2019) of broad-spectrum, beta-lactamase-sensitive penicillin and the prevalence of type 2 diabetes (T2D) (2019).

Datasheets and diagrams were developed to present the positive or negative associations (**Tables 1**–**3** and **Figures 1**–**7**) between the consumption of certain antibiotic types and the prevalence of T1D and T2D in the European countries featured in the study.

**Table 2:** Variance analysis (ANOVA) of the rank order of type 1 diabetes (T1D) (top 10, shaded) compared to the rank order of antibiotic consumption with possible “enhancing” [tetracycline (J01A): *p* = 0.015; narrow spectrum, beta-lactamase-sensitive penicillin (J01CE): *p* = 0.008] or “inhibiting” [broad-spectrum, beta-lactamase resistant, combination penicillin (J01CR): *p* = 0.005; quinolone (J01M): *p* = 0.036] effects on the prevalence of T1D.

Countries	T1D	Countries	J01A	Countries	J01CE	Countries	J01CR	Countries	J01M
Finland	**130.21**	UK	**27.35**	Denmark	**28.34**	Portugal	35.07	Cyprus	17.83
Sweden	**86.24**	Iceland	25.27	Sweden	**27.59**	Italy	33.87	Hungary	16.29
Norway	**71.19**	Finland	**24.72**	Norway	**21.56**	Spain	31.95	Bulgaria	14.6
Ireland	**68.48**	Netherlands	**23.72**	Slovenia	**14.33**	Malta	29.95	Italy	14.42
UK	**58.24**	Sweden	**22.36**	Czechia	**11.17**	Croatia	28.86	Romania	12.92
Denmark	**53.89**	Latvia	20.46	Iceland	10.93	Austria	26.75	Spain	12.79
Netherlands	**42.79**	Norway	**19.59**	Finland	**7.88**	Hungary	25.62	Portugal	12.12
France	**42.01**	Germany	**16.26**	Austria	6.58	Cyprus	24.84	Malta	11.94
Germany	**39.82**	Estonia	15.41	Germany	**6.26**	Slovenia	24.4	Luxembourg	11.42
Czechia	**38.44**	Ireland	**14.46**	Slovakia	6.07	Luxembourg	24.16	Austria	10.16
Estonia	**37.8**	France	**13.55**	Ireland	**5.34**	Romania	23.83	Belgium	10.02
Hungary	**36.05**	Cyprus	12.68	UK	**4.77**	Belgium	22.92	Slovakia	9.73
Belgium	**35.71**	Czechia	**12.68**	Croatia	3.81	Ireland	**20.92**	Germany	**9.63**
Lithuania	**35.7**	Poland	11	Romania	3.1	France	**19.75**	Slovenia	9.38
Austria	**33.74**	Denmark	**10.79**	Netherlands	**2.75**	Slovakia	18.32	Latvia	8.88
Cyprus	**33.63**	Lithuania	10.57	Hungary	1.9	Czechia	**17.3**	Greece	8.43
Poland	**33.23**	Bulgaria	9.62	Estonia	1.85	Greece	16.25	Croatia	8.31
Spain	**33.2**	Belgium	9.07	Lithuania	1.65	Poland	14.25	Netherlands	**8.15**
Luxembourg	**33.1**	Hungary	8.66	Poland	1.14	Iceland	14.13	Estonia	**8.09**
Malta	**33.1**	Luxembourg	8.26	Bulgaria	1.06	Estonia	13.65	France	**6.92**
Croatia	**31.28**	Slovakia	8.08	France	**0.72**	Latvia	12.11	Lithuania	6.83
Iceland	**29.7**	Greece	7.98	Malta	0.51	Netherlands	**11.22**	Poland	6.31
Greece	**29.46**	Austria	7.41	Spain	0.45	Bulgaria	11.08	Czechia	**5.7**
Slovenia	**28.88**	Malta	6.9	Latvia	0.44	Lithuania	10.14	Sweden	**5.55**
Italy	**26.39**	Croatia	6.04	Cyprus	0.3	**Finland**	**5.4**	Finland	**4.83**
Slovakia	**25.67**	Spain	5.2	Greece	0.25	**Denmark**	**4.9**	Iceland	4.78
Portugal	**24.38**	Portugal	4.87	Belgium	0.13	**UK**	**4.54**	Ireland	**4.18**
Bulgaria	**15.6**	Romania	4.19	Portugal	0.11	**Germany**	**1.87**	Denmark	**3.17**
Latvia	**15.56**	Slovenia	3.49	Luxembourg	0.09	**Sweden**	**1.79**	Norway	**2.96**
Romania	**14.35**	Italy	2.48	Italy	0	**Norway**	**0.12**	UK	**2.61**

Significant concordance was observed between countries with higher prevalence and T1D and the higher consumption of “enhancer” antibiotics. Similar concordance was found between the higher prevalence rate of T1D and the low consumption of “inhibitor” antibiotics.

Bold indicate the identical countries between the rank order columns of T1D, T2D and the rank orders of antibiotic consumption.

**Table 3:** Variance analysis (ANOVA) of the rank order of type 2 diabetes (T2D) (top 10, shaded) compared to the rank order of antibiotic consumption with possible “enhancing” [cephalosporin (J01D): *p* = 0.084; quinolone (J01M): *p* = 0.054] or “inhibiting” [broad-spectrum, beta-lactamase-sensitive penicillin (J01CA): *p* = 0.012] effects on the prevalence of T1D.

Countries	T2DM	Countries	J01D	Countries	J01M	Countries	J01CA
Germany	**11,441.28**	Romania	30.59	Cyprus	**17.83**	Lithuania	35.25
Portugal	**10,628.7**	Greece	24.39	Hungary	**16.29**	France	31.18
Spain	**7,750.86**	Slovakia	**22.99**	Bulgaria	14.6	Latvia	25.94
Cyprus	**7,719.05**	Malta	**22.01**	Italy	14.42	Belgium	22.2
Czechia	**7,675.09**	Germany	**21.74**	Romania	12.92	Spain	**21.94**
Austria	**7,214.85**	Cyprus	**20.53**	Spain	**12.79**	Denmark	21.12
Hungary	**7,051.25**	Bulgaria	19.13	Portugal	**12.12**	UK	20.43
Slovakia	**6,922.78**	Croatia	16.39	Malta	**11.94**	Slovenia	19.36
Finland	**6,767.06**	Luxembourg	15.3	Luxembourg	11.42	Romania	17.97
Malta	**6,702.58**	Hungary	**13.95**	Austria	**10.16**	Bulgaria	17.51
Romania	**6,553.33**	Finland	**13.54**	Belgium	10.02	Germany	**17.27**
Denmark	**6,467.17**	Poland	13.49	Slovakia	**9.73**	Iceland	16.97
Bulgaria	**6,275.16**	Austria	**12.91**	Germany	**9.63**	Finland	**16.84**
Poland	**6,182.76**	Czechia	**11.17**	Slovenia	9.38	Estonia	16.78
Italy	**6,052.39**	Estonia	10.73	Latvia	8.88	Poland	16.36
Netherlands	**5,973.78**	Italy	10.69	Greece	8.43	Ireland	14.92
Slovenia	**5,895.55**	Spain	**9.4**	Croatia	8.31	Luxembourg	14.29
Greece	**5,834.31**	Portugal	**9**	Netherlands	8.15	Greece	14.17
Norway	**5,477.75**	Lithuania	8.63	Estonia	8.09	Norway	13.98
Latvia	**5,408.46**	France	8.62	France	6.92	Netherlands	13.87
Iceland	**5,405.2**	Belgium	6.29	Lithuania	6.83	Italy	12.02
France	**5,354.63**	Ireland	5.95	Poland	6.31	Croatia	11.37
Sweden	**5,226.82**	Latvia	4.8	Czechia	5.7	Portugal	**9.63**
Croatia	**5,078.91**	Iceland	3.04	Sweden	5.55	Cyprus	**9.12**
Belgium	**4,887.57**	Slovenia	2.81	Finland	4.83	Sweden	8.73
Luxembourg	**4,733.18**	UK	1.86	Iceland	4.78	Czechia	**6.97**
Estonia	**4,437.15**	Sweden	1.14	Ireland	4.18	Austria	**6.58**
Lithuania	**4,080.3**	Norway	0.59	Denmark	3.17	Hungary	**6.46**
UK	**3,992.3**	Netherlands	0.42	Norway	2.96	Slovakia	**5.02**
Ireland	**3,075.52**	Denmark	0.19	UK	2.61	Malta	**2.54**

Non-significant concordance was observed between countries with higher prevalence of T2D and the higher consumption of “enhancer” antibiotics. Similar concordance was found between the higher prevalence rate of T2D and the low consumption of “inhibitor” antibiotics.

T2DM, type 2 diabetes mellitus.

Bold indicate the identical countries between the rank order columns of T1D, T2D and the rank orders of antibiotic consumption.

## Results

A positive significant correlation was estimated between the consumption of tetracycline (J01A) and the prevalence of T1D (Pearson’s *r* = 0.58, *p* = 0.001), similarly to the consumption of narrow-spectrum, beta-lactamase-sensitive penicillin (J01CE; Pearson’s *r* = 0.488, *p* = 0.006) and narrow-spectrum, beta-lactamase-resistant penicillin (J01CF; Pearson’s *r* = 0.43, *p* = 0.018) and the prevalence of T1D. Inverse (negative) significant correlation was found between the prevalence of T1D and the utilization of broad-spectrum, beta-lactamase-resistant combination penicillin (J01CR; Pearson’s *r* = −0.519, *p* = 0.003), macrolides (J01F; Pearson’s *r* = −0.473, *p* = 0.008), and quinolone (Pearson’s *r* = −0.558, *p* = 0.001). A non-significant negative correlation was recorded between the consumption of cephalosporin (J01D) and the prevalence of T1D (Pearson’s *r* = −0.349, *p* = 0.056) (**Table 1** and **Figures 1**–**4**).

The prevalence of T2D showed a positive significant association with the consumption of cephalosporin (Pearson’s *r* = 0.364, *p* = 0.048) and quinolone (Pearson’s *r* = 0.41, *p* = 0.025). A non-significant negative correlation was found between the prevalence of T2D and the consumption of broad-spectrum, beta-lactamase-sensitive penicillin (J01CA; Pearson *r* = −0.339, *p* = 0.067) (**Table 1** and **Figures 5**–**7**).

ANOVA compared the rank order of T1D (top 10, shaded part in **Table 2**) to the rank order of consumption of antibiotics with possible “enhancing” [tetracycline (J01A): *p* = 0.015; narrow-spectrum, beta-lactamase-sensitive penicillin (J01CE): *p* = 0.008] or “inhibiting” [broad-spectrum, beta-lactamase-resistant, combination penicillin (J01CR): *p* = 0.005; cephalosporin (J01D): *p* = 0.036; quinolone (J01M): *p* = 0.003] effects on the prevalence of T1D. The results indicated that countries with a higher consumption of “enhancing” antibiotics and low consumption of “inhibiting” antibiotics experience a higher prevalence of T1D.

A similar comparison of the prevalence of T2D (**Table 3**) revealed that the higher consumption of “enhancing” antibiotics (cephalosporin and quinolone) and the low utilization of broad-spectrum, beta-lactamase sensitive penicillin (J01CA) with “inhibitor” effects were associated with a higher prevalence rate of T2D.

The OR calculations clearly supported the results of Pearson’s correlation, indicating the higher risk of the development of T1D in populations consuming tetracycline (J01A) and narrow-spectrum penicillin (J01CE, J01CF), while broad-spectrum antibiotics [broad-spectrum, beta-lactamase-resistant combination penicillin (J01CR), cephalosporin (J01D), macrolides (J01F), and quinolone (J01M)] exhibited inhibitory effects on the development of T1D. Similarly, the risk of the development of T2D was higher among populations consuming significantly higher amounts of broad-spectrum antibiotics [broad-spectrum, beta-lactamase-sensitive penicillin (J01CR), cephalosporin (J01D), and quinolone (J01M)].

**Figures 1**–**7** display the graphical appearance of the positive and negative associations between the consumption of different antibiotic classes and the prevalence of T1D and T2D.

## Discussion

Genetic analysis of T1D has found 50 susceptibility regions and the major pathways contributing to increased risk of T1D, with some other loci shared across immune disorders. The genetic factors considered as major risks for the development of T1D were identified, as follows: the T1D-associated single nucleotide polymorphisms (SNPs) in the HLA gene and, more specifically, the HLA-DQ and HLA-DR protein-coding genes *DQA1* and *DQB1* (20). Researchers agree that the development of T1D cannot be explained using only the genetic background. Several external factors must be considered, including different infections, the intestinal microbiota, vaccines, vitamin D3 deficiency, breastfeeding, and dietary factors, among others (4, 12).

Even the geographical location of a country (lassitude and longitude), cold climate, etc., were considered as causative factors. The dramatic increase of the prevalence of T1D after World War II (WWII) contradicts all such concepts because the geographical and meteorological conditions were the same before WWII, but the extensive discovery and utilization of antibiotics, beginning with penicillin, started only after WWII. In addition, the outstanding use of penicillin is still observed recently in the Scandinavian countries, along with the highest prevalence of T1D.

The most important diagnostic criteria for T1D are elevated blood glucose levels (hyperglycemia) and the presence of autoantibodies, both of which occur or are present even before the development of beta cell destruction. Autoantibodies were observed against insulin (IAA), glutamic acid decarboxylase (GADA), insulinoma-associated autoantigen 2 (IA2A), and/or zinc transporter 8 (ZnT8A) and may occur many years before the onset of symptoms (21).

Appropriate composition and diversity are necessary for the development of a normally functioning immune system. Several studies indicated that the gut microbial composition differs between healthy hosts and patients with T1D or at risk of T1D.

Animal experiments using germ-free (GF) and gnotobiotic mouse models demonstrated their important role in the alteration and the differentiation of natural immune cell types, particularly interleukin 17 (IL-17)-producing CD4^+^ T cells (T helper 17, Th17) and Foxp3^+^ regulatory T cells (Tregs) (22, 23). It was observed that segmented filamentous bacteria (SFB) induce the expression of pro-inflammatory Th17 cells, which play important roles in maintaining the mucosal barrier and preventing non-obese diabetic (NOD) mice from developing T1D (24, 25). In other mouse models of autoimmune disease (e.g., K/BxN mouse model of autoimmune arthritis), SFB was shown to induce the disease progression through augmented Th17 accumulation, which suggests that their role in the development of autoimmunity is etiologically specific (26). The role of “protective” bacteria, such as *Lactobacillus*, *Bifidobacterium*, and *Clostridium* species, has been shown to be implicated in the induction of anti-inflammatory Tregs. Other bacteria, such as *B. fragilis* polysaccharide A (PSA), induce IL-10 production, which suppresses the responses of Th17 cells (27, 28).

Experimental observations indicated a significant reduction in the abundance of *Lactobacillus*, *Bryantella*, *Bifidobacterium*, and *Turicibacter* in bio-breeding diabetes-prone (BB-DP) rats, while the abundances of *Bacteroides*, *Eubacterium*, and *Ruminococcus* increased in BB-DP rats compared with those in bio-breeding diabetes-resistant (BB-DR) rats (29–32).

In pediatric diabetes, the abundances of Actinobacteria and Firmicutes and the Firmicutes-to-Bacteroidetes ratio were significantly decreased, while the bacterial number of Bacteroidetes significantly increased in healthy children. In diabetic children (T1D), a marked decrease of *Lactobacillus* and *Bifidobacterium* was observed, which showed an association with higher blood glucose levels (33–36).

A large number of publications provided evidence for the possible role of the gut microbiota in metabolic diseases, including T2D.

A major meta-analysis of the relevant literature indicated the association of T2D and some specific taxa of the microbiome (37). It has been consistently reported that the genera *Bifidobacterium*, *Bacteroides*, *Faecalibacterium*, *Akkermansia*, and *Roseburia* were reduced in T2D, while the abundances of *Ruminococcus*, *Fusobacterium*, and *Blautia* markedly increased in T2D. A high level of intestinal permeability was observed in T2D as well, which facilitates the translocation of bacterial products in the blood, resulting in metabolic endotoxemia (38).

The alteration of the gut flora has been frequently associated with metabolic disorders, such as diabetes mellitus, insulin resistance, obesity, and T2D. Low-grade inflammation with elevated IL-6 was also observed in the presence of certain Gram-negative bacteria, including *Prevotella copri* and *Bacteroides vulgatus* (39).

The role of dysbiosis in the occurrence of T1D and T2D has been widely discussed and documented in the relevant scientific literature (referred to above). We discovered that the different antibiotic consumption patterns in European countries showed strong (positive/negative) statistical correlations with the prevalence of T1D and T2D, probably through the modification of the gut microbiome. It was observed that countries (mostly Scandinavian) with higher consumptions of T1D “enhancing” antibiotics, e.g., tetracycline (J01A) and narrow-spectrum penicillin (J01CE, J01CF), experience a higher prevalence of T1D, along with the low consumption of “inhibitor” antibiotics, e.g., broad-spectrum, beta-lactamase-resistant, combination penicillin (J01CR) and other broad-spectrum antibiotics (macrolides or quinolone).

According to our previous observations, we found it interesting to learn that the higher consumption of the same antibiotics (tetracycline or narrow-spectrum penicillin) showed an “enhancer” relationship with seemingly unrelated diseases. A higher incidence of some major carcinomas was also observed in countries with higher consumptions of tetracycline and narrow-spectrum penicillin (40). The phenomenon that patients with multiple sclerosis have a three to fivefold higher chance of developing T1D had been documented in the literature (41–43).

We have previously observed that the same antibiotics (tetracycline and narrow-spectrum penicillin) also showed a positive significant correlation with the prevalence of multiple sclerosis (44). Based on the statistical comparison of large, publicly available databases on antibiotic consumption and the prevalence of T1D and T2D in 30 European countries, we found convincing statistical associations between the prevalence of T1D and T2D and the use of different antibiotic classes exhibiting “enhancer” and “inhibitor” potency. The rank order of countries with the first highest prevalence of T1D and T2D showed concordance with that of countries with the highest consumption of “enhancer” and the lowest consumption of “inhibitor” classes of antibiotics (**Tables 2** and **3**). It is suspected that the different classes of antibiotics, the production of different types of dysbiosis in the gut flora, and the changes in the the composition and production of mediator molecules produced by different microbial taxa play a part in the enhancer or inhibitor factors in the development of T1D and T2D, probably through the gut-brain axis (GBA) or other molecular mechanisms.

Our findings might initiate further research regarding these associations, which could identify new molecules (drugs) relevant to reducing the occurrence of diabetes.

### Limitations

The major limitation of our study is that the effects of antibiotics, outlined above, could not be proven at the individual level and could not be interpreted as the direct effect of antibiotics, only as a possible triggering factor inducing dysbiosis, which might have enhancing or inhibiting effects on the development of diabetes through mediator molecules and GBA.

However, the strength of this study is its comparison of large databases, which provides an appropriate background for valid results. The statistical correlations (Pearson) are consistent with the results of ANOVA.

## Data Availability Statement

The original contributions presented in the study are included in the article/supplementary material. further inquiries can be directed to the corresponding author.

## Author Contributions

GT: developing the concept, writing the manuscript. MN: calculating statistics and designing tables and diagrams. MR: collecting and evaluating the literature included in the study. LB: critical review of the manuscript and corrections. All authors contributed to the article and approved the submitted version.

## Funding

This research did not receive any specific grant from funding agencies in the public, commercial, or not-for-profit sectors.

## Conflict of Interest

The authors declare that the research was conducted in the absence of any commercial or financial relationships that could be construed as a potential conflict of interest.

## Publisher’s Note

All claims expressed in this article are solely those of the authors and do not necessarily represent those of their affiliated organizations, or those of the publisher, the editors and the reviewers. Any product that may be evaluated in this article, or claim that may be made by its manufacturer, is not guaranteed or endorsed by the publisher.
